# eHealth literacy in a migrant community and its association with chronic disease

**DOI:** 10.3389/fpubh.2025.1668262

**Published:** 2025-09-29

**Authors:** Madalena Garcia, Rosa Machado, Isaura Serra, Ana Lúcia João

**Affiliations:** ^1^Escola Superior de Saúde, Instituto Politécnico de Setúbal, Setúbal, Portugal; ^2^The Local Health Unit Arrábida E.P.E., Setúbal, Portugal; ^3^Universidade de Évora, Escola Superior de Enfermagem S. João de Deus, Évora, Portugal; ^4^Universidade de Évora, Comprehensive Health Research Centre (CHRC), Évora, Portugal; ^5^Higher School of Health of Santarém, Santarém Polytechnic Institute, Santarém, Portugal

**Keywords:** eHealth, health literacy, migrant health, emigrants and immigrants, social determinants of health, chronic disease, eHLQ

## Abstract

**Introduction:**

eHealth literacy or digital health literacy has been widely recognized for its impact on health outcomes. Migrant populations face additional challenges related to low literacy, social vulnerability, and health frailty, which may also indicate reduced levels of digital health literacy. The aim of this study was to assess digital health literacy levels in a migrant population and to examine their relationship with sociodemographic characteristics and health-related variables.

**Methods:**

This descriptive, cross-sectional, and exploratory study used a convenience sample of 101 migrant residents from a neighborhood in the Lisbon Metropolitan Area who were fluent in Portuguese. Non-Portuguese speakers were excluded, which may limit representativeness. The small sample size and the non-probabilistic recruitment strategy also constrain the generalizability of findings. Data collection included a sociodemographic and health questionnaire and the validated Portuguese version of the eHealth Literacy Questionnaire.

**Results:**

Participants reported generally low levels of eHealth literacy. Statistically significant differences were found between individuals with and without chronic disease: those without chronic disease obtained higher scores in most eHLQ dimensions. Associations were also observed with age and educational level. Effect sizes indicated that these associations ranged from weak (e.g., access to digital services that work, r = 0.286) to moderate (e.g., ability to actively engage with digital services, r = 0.472; digital services that suit individual needs, r = 0.432), providing a clearer picture of the magnitude of effects.

**Discussion:**

The findings show that despite fluency in Portuguese, migrants demonstrated persistent barriers to effectively using digital health tools, particularly those living with chronic conditions. These results highlight the intersection of clinical vulnerability and digital exclusion in this population.

**Conclusion:**

This exploratory study, while limited by the exclusion of non-Portuguese speakers, convenience sampling, and a small sample size, contributes valuable evidence on digital health inequalities in migrant communities. Effect sizes indicate that associations between eHL and perceived health status or chronic disease are of small to moderate magnitude, underlining the urgent need for culturally sensitive interventions, targeted training, and policy measures to reduce digital health disparities.

## 1 Introduction

The concept of health literacy (HL) was initially defined by the World Health Organization (WHO) in 1998 and later updated in 2021. Currently, HL is understood as “the knowledge and personal skills acquired through daily activities, social interactions, and across generations (…) that enable individuals to access, understand, appraise, and use information and services to promote and maintain good health and well-being for themselves and those around them” (1). In addition to the conceptual definition, there is also a pressing need to provide resources that support the development and practical application of these skills.

With the technological advances of recent decades and the growing presence of digital tools in the health sector, the concept of eHealth literacy (eHL) emerged, formalized by Norman and Skinner in 2006 (2). In general, eHL represents an adaptation of HL to the digital context, that is, to the access and use of information mediated by technologies. Following this, the Lily model was proposed, the first theoretical model of eHL, which distinguishes two main groups: analytic literacy and context-specific literacy, each subdivided into six secondary domains. Analytic literacy includes traditional, media, and information literacy, while context-specific literacy encompasses computer, science, and HL.

Since its initial formulation, the concept of eHL has evolved alongside technological developments, incorporating new variables associated with emerging challenges and opportunities. From a more operational perspective, Chan and Kaufmann sought to translate the Lily model into practical applications, highlighting the importance of considering social, emotional, and cultural factors, as well as individual motivation and attitudes toward technology (3). In this context, individual decision-making mechanisms have also been identified as influential in shaping how people access and use digital health information.

Norman acknowledged the growing diversity of Internet uses and the widespread adoption of mobile devices, noting that increased access to digital health information has not always been accompanied by a corresponding improvement in the quality and reliability of the information available (4, 5). This reality has reinforced the importance of individual skills to critically assess, select, and use information—an essential aspect in the era of information overload (infodemic) (6).

Subsequently, other authors proposed the inclusion of new dimensions within the concept of eHL, such as communication skills, bodily experience (7), concerns about data privacy and security, and the lack of motivation (8).

In an effort to systematize and deepen the concept, Norgaard et al. proposed the eHL Framework (eHLF) (9), which considers the interdependence between the digital health system and users’ individual competencies. This model organizes eHL into seven domains, distributed across two main axes: the horizontal axis, which ranges from individual competencies to dependence on the functioning of digital systems; and the vertical axis, which shifts between observable behaviors and more subjective dimensions, such as attitudes and perceptions. Thus, the model encompasses both the individual’s ability to process information and engage with their own health, and the effectiveness of digital health systems in supporting the safe, personalized, and motivated use of their services.

More recently, Paige introduced the “Transactional Model of eHealth Literacy” (10), which deepens the contextual and dynamic components of eHL. In this model, eHL is defined as “the ability to locate, understand, exchange, and evaluate health information in online environments while accounting for dynamic contextual factors, and to apply the acquired knowledge across different ecological levels with the aim of maintaining or improving health” (p. 9).

The importance of eHL in health outcomes has been increasingly recognized. Several studies have demonstrated the relationship between eHL and indicators such as quality of life, health promotion, mental health, chronic disease management, and overall health status (11). In the field of health promotion, behaviors such as individual responsibility for health, self-actualization, and social support stand out as practices associated with eHL. In addition, several determinants of eHL have been identified, with education and training emerging as the most relevant factors, followed by social support, although the latter shows a less significant impact (11).

Indeed, these findings reinforce the need to consider eHL as a key determinant for health promotion in increasingly digitalized societies. More recently, the literature has proposed a broader perspective, positioning it as a super-determinant of health. In this regard, the study conducted by Andersson and Gonzalez (12), by integrating social and cultural determinants as well as structural dimensions such as education, access to technological infrastructures and socioeconomic conditions, expands the classical understanding of eHL. From this perspective, it becomes evident that eHL does not operate in isolation; rather, it interacts with and amplifies other social determinants of health, thereby directly influencing equity in both access to healthcare and health outcomes.

In migratory contexts, where linguistic, institutional, and cultural barriers may coexist, this approach proves particularly relevant, as the promotion of eHL requires policies and strategies that combine individual empowerment with structural interventions aimed at digital and social inclusion (12).

On the other hand, the strategic importance of eHL is also evident at the international level. The World Health Organization’s *Global Strategy on Digital Health 2020–2025* (13), emphasizes that digital transformation in health must be grounded in the principles of equity, accessibility, and individual empowerment. Similarly, the European Commission has advanced several convergent initiatives, notably the *EU4Health Program (2021–2027)*, which incorporates digitalization as a central pillar for building more resilient health systems, and the *Action Plan for Integration and Inclusion (2020)*, which advocates for the development of user-centered public digital services that are inclusive and responsive to cultural diversity (14).

In parallel, the European Economic and Social Committee (EESC) has recommended the establishment of a comprehensive European strategy for digital health literacy, highlighting the need to actively engage vulnerable groups in the development of digital solutions (15). This underscores the growing political attention to digital health literacy as a fundamental pillar in reducing health inequalities.

The most vulnerable groups tend to have the lowest levels of HL, particularly individuals with disadvantaged socioeconomic conditions, low educational attainment, or advanced age (16).

With the increasing digitalization of healthcare and society in general, and despite the enormous potential of digital technologies to facilitate access to health services, promote healthy behaviors, and improve disease management, digital exclusion has become a significant concern. There is, therefore, an increased risk of further marginalizing individuals with low levels of digital eHL (3, 7, 17, 18).

According to the most recent data from the International Organization for Migration (IOM, 2024), there were approximately 281 million international migrants in 2020, representing about 3.6% of the global population (19). The number of African migrants has increased significantly over recent decades, with around 11 million of the 19.5 million recorded currently residing in Europe.

In addition to access to digital devices, individuals need an internet connection to benefit from digital health solutions (17–20). This access can be influenced by social, economic, and cultural factors, such as assigned social roles, income, or employment status. In vulnerable contexts, these limitations may widen existing inequalities or even create new forms of health exclusion, particularly among migrant populations (21). These barriers represent a growing public health concern, as they exacerbate structural inequalities and undermine equity in access to healthcare. Although vulnerability is often associated with age, socioeconomic status, and educational level, several authors also highlight migrant status as an additional determining factor (11).

In the specific case of these populations, such issues become even more pressing. Recent studies have shown that migrants face additional barriers in using digital health services, not only related to digital proficiency but also due to linguistic, cultural, and institutional obstacles (12).

The social and cultural dimension is therefore inseparable from the broader concept of HL and eHL, and should be explicitly considered in the assessment and planning of interventions in this area. Evaluating eHL therefore requires a contextualized approach that takes into account the cultural, linguistic, and social realities of the populations involved (7, 22).

Within this framework, the present study aimed to assess eHL in a migrant community, considering participants’ sociodemographic characteristics, their self-perceived health status, and the presence of chronic disease.

## 2 Materials and methods

### 2.1 Study design

This quantitative study followed a descriptive and correlational approach with a cross-sectional and exploratory design. The study does not report causal relationships but rather highlights trends and associations; therefore, the findings are not intended to support causal inferences. Data were collected using a questionnaire composed of two sections. The first section included sociodemographic and professional information, as well as relevant health history. The second section comprised the official Portuguese version of the “eHealth Literacy Questionnaire” (eHLQ) (23).

It is important to explicitly recognize the limitations inherent to this design. The cross-sectional nature of the study prevents the establishment of causal relationships between variables, and the analysis may be affected by confounding bias, given that other unmeasured factors could have influenced the observed associations. Consequently, the results should be interpreted with caution, acknowledging that they identify correlations and patterns but not cause-effect relationships.

### 2.2 Population and sample

The target population of this study consisted of migrant individuals residing in a neighborhood in the Lisbon Metropolitan Area, who were either native speakers of Portuguese or fluent in the language. The inclusion of migrant participants aimed to address the need to deepen knowledge about eHL in socially vulnerable groups, while ensuring adequate comprehension of the instruments used in the study. Therefore, proficiency in either Portuguese or English was established as an inclusion criterion to ensure proper comprehension of the instruments and the validity of the data collected. However, all participants recruited were either native or fluent Portuguese speakers.

The study sample consisted of 101 participants, representing 17.7% of the adult population living in the community, estimated at approximately 570 people. A 95% confidence level and a 9% margin of error were reported; however, these values were not derived from an *a priori* sample size estimation but calculated retrospectively. As such, they should not be interpreted as an indicator of statistical power or adequacy of the sample design. The absence of a priori sample size estimation constitutes a limitation of this study, as it does not allow for a robust justification of statistical power. This limitation should be considered when interpreting the results, as the relatively small sample size and the convenience sampling strategy reduce generalizability.

The sample distribution was balanced, with 53.5% women and 46.5% men, with no statistically significant differences between sexes. The majority of participants were of working age (88.1%), with 12.9% aged between 18 and 24 years and 75.2% between 25 and 64 years. Only 11.9% were aged 65 or older.

Most participants (92.1%) were born outside Portugal, with only 8 individuals (7.9%) born in the country but of foreign descent, in accordance with the predefined inclusion criteria. Participants reported four countries of birth: Gabon, Equatorial Guinea, Cape Verde, and São Tomé and Príncipe. There was a predominance of individuals from São Tomé and Príncipe (50.5%), followed by Cape Verde (39.6%), Portugal (7.9%)—with Cape Verdean or Santomean ancestry—and a small minority from Gabon and Equatorial Guinea (2%). The majority of participants reported having regular legal status in Portugal (67.3%), while others were undocumented (8.9%) or in the process of regularization (23.8%).

Regarding educational attainment, most participants (71.3%) had not completed secondary education, and only a small minority (2%) held a higher education degree. Notably, 9.9% of participants had no formal education.

In terms of employment status, 32.7% of respondents were employed full-time, followed by 29.7% who were unemployed, 17.8% working part-time, 9.9% retired, 5% engaged in other types of informal work, and 4% were students.

### 2.3 Sampling and eligibility criteria

Participant selection was carried out through non-probabilistic convenience sampling, based on the accessibility of individuals during the data collection period. Eligible participants included migrant residents and their direct descendants living in a neighborhood in the Lisbon Metropolitan Area, aged 18 years or older, who expressed willingness to participate voluntarily and who provided written informed consent after receiving clarification about the study’s objectives and procedures.

Individuals who did not meet the inclusion criteria or who were unable to understand the basic instructions of the questionnaires or to express their answers intelligibly in Portuguese or English were excluded, as this would have prevented reliable data collection, compromised the validity of the responses, and raised ethical concerns regarding informed consent and proper understanding of the study.

### 2.4 Data collection procedures and instruments

Data collection took place between December 3 and December 10, 2022, through the administration of paper-based questionnaires, completed in person under the supervision of the researcher. This methodological choice followed the official guidance of the eHLQ, which recommends oral administration to ensure participation of individuals with low literacy or reading difficulties, to reduce the risk of exclusion. Participants were also given the option to complete the questionnaire autonomously, asking for support only if needed. Participants were invited to take part in the study during outreach visits to the neighborhood, carried out at various times throughout the week, including weekends and public holidays, except during nighttime hours. This approach aimed to ensure greater accessibility and representativeness of the sample.

Community engagement was ensured through a partnership with a non-profit social welfare institution with a longstanding daily presence in the neighborhood, as well as through prior contact with the local residents association. The data collection was disseminated both within the community and through their online communication channels and was conducted door-to-door. The researchers were accompanied by volunteer members of the residents association, which contributed to building trust and facilitating participation.

The data collection instrument consisted of a structured questionnaire with two main sections. The first section addressed the sociodemographic characterization of participants. The following variables were collected: age (in full years), gender (male/female/other), marital status (single, married/civil partnership, separated/divorced, widowed), education level (no formal education, primary, lower secondary, upper secondary, higher education), employment status (employed, unemployed, student, retired, informal work/other), length of residence in Portugal (in years), and documentation status (regular, irregular, or in the process of regularization).

In terms of health, two variables were assessed: self-reported health status (on a scale from 0 = very poor to 10 = very good) and the presence of chronic disease. The latter was operationalized through a direct question (“Do you have any chronic health problems diagnosed by a health professional?”), supplemented by a predefined list of common conditions (hypertension, diabetes, asthma/chronic respiratory disease, cardiovascular disease, and others), with the option of open responses for unlisted conditions.

The second section included the eHLQ, developed by Norgaard et al. (9), and administered in its official Portuguese version, validated by Kayser et al. (23). This second-generation instrument was designed to assess eHL within a constantly evolving context, characterized by the increasing use of digital technologies and social media in the healthcare field (24).

The eHLQ consists of seven dimensions that evaluate engagement with digital health technologies across the dimensions: “Using technology to process health information” Understanding of health concepts and language,” “Ability to actively engage with digital servisses,” “Feel safe and in control”; “Motivate to engage with digital services”; “Access to digital services that work, Digital services that suit individual needs”.

The instrument has demonstrated excellent psychometric properties (23, 25, 26) when compared to other similar scales (20). Responses are recorded on a 4 point Likert scale, ranging from 1 – Strongly disagree to 4 – Strongly agree. The eHLQ is considered to have superior psychometric properties relative to other existing instruments with similar objectives.

The internal consistency of the instrument was calculated in the present sample, yielding an overall Cronbach’s alpha of 0.95. For the seven dimensions, the coefficients ranged from 0.60 to 0.92, indicating internal reliability from acceptable to excellent in the specific context of this migrant population.

#### 2.4.1 Ethical considerations

All ethical considerations inherent to the different stages of the research were duly observed, in accordance with the principles of the Declaration of Helsinki (27), particularly concerning voluntary participation and data protection. Ethical approval for the study was granted by the Regional Health Authority of Lisbon and Tejo Valley.

It is also important to highlight that the use of the eHLQ was subject to licensing. Following initial consent from the corresponding author, obtained electronically, formal authorization for its use was secured through a licensing agreement between Swinburne University of Technology and the School of Health Sciences of the Polytechnic Institute of Setúbal.

#### 2.4.2 Statistical analysis

Descriptive statistics were used to summarize the sociodemographic and health characteristics of participants, as well as the distribution of eHLQ scores across the seven dimensions. Given the exploratory nature of the study, the analysis was primarily oriented toward identifying patterns and associations, rather than establishing causal relationships.

Before conducting parametric tests, we assessed the assumptions of normality, homogeneity of variances, and linearity. Normality was evaluated using the Shapiro–Wilk test and inspection of Q–Q plots. Homogeneity of variances was tested with Levene’s test, and linearity was verified through scatterplots of the variables involved.

For bivariate analyses, we applied Student’s t-tests to compare mean eHLQ scores between two groups, and one-way ANOVA for comparisons across more than two groups. When ANOVA results were significant, Tukey’s *post hoc* test was conducted. Pearson’s correlation coefficients were used to explore associations between eHLQ dimensions and continuous variables. Alongside statistical significance (*p* ≤ 0.05), effect sizes were reported: Cohen’s d for t-tests, eta squared (η^2^) for ANOVA, and correlation strength (r) for Pearson’s tests, thereby allowing for interpretation of the practical magnitude of the results.

All analyses were conducted using IBM SPSS Statistics for Windows, version 29.

## 3 Results

### 3.1 Self-perceived health and chronic disease history

Participants’ perceptions of their own health produced an average score of 6.69 on a scale from 0 (“very poor”) to 10 (“very good”). Migrants with irregular legal status reported the highest self-assessed health, with an average score of 8 on the same scale. Length of stay in the country showed no statistically significant differences in self-perceived health; however, a declining trend was observed after 5 years of residence. Notably, among those who had been living in Portugal for less than 1 year, approximately two-thirds rated their health between 8 and 10, remaining above the overall sample average.

As shown in Table 1, almost half of the sample (45.5%) reported at least one chronic disease, with hypertension being the most common (25.7%). This data highlights the high health vulnerability of the community analyzed and is a critical factor in interpreting levels of digital health literacy.

**Table 1:** Prevalence of chronic diseases among migrant participants (*n* = 101).

Chronic disease	*n*	%
Do you have any chronic disease?	46	45.5
Diabetes	14	13.9
High blood pression	26	25.7
Asthma	3	3.0

Regarding the perceived need for healthcare, 68% of participants reported having felt the need for healthcare in the previous year, while 18% stated that they had not felt the need for care.

In terms of healthcare service utilization, most participants sought care through primary healthcare services (55.4%), followed by hospital emergency services (16.8%). A considerable proportion (10.9%) reported being unable to access healthcare when needed. Private healthcare services were used by a smaller segment of the sample (3%), while a minority (2%) acknowledged not seeking care despite having felt the need for it.

In the overall sample, 31.7% (*n* = 32) had never accessed healthcare services in Portugal. Among these participants, the reasons included being unable to access care (*n* = 11), not having felt the need for care (*n* = 19), or not seeking care despite needing it (*n* = 2). Within this group, the majority (*n* = 20) reported not having regular legal status in the country (*n* = 9, *n* = 10, and *n* = 1, respectively).

Among the reasons reported for the inability to access healthcare, the most frequently cited was the lack of a national health service user number (reported by 9.9% of the total sample), which, with the exception of one participant, was selected by all those who experienced difficulties accessing healthcare services. The next most common reason was migration status (8.9% of the total sample), also selected by nearly all respondents who answered this question. Other reported barriers included financial constraints (5%), difficulty traveling (3%), and lack of knowledge on how to proceed (3%). Notably, fear and language barriers were not selected by any participant.

### 3.2 eHealth literacy

The average scores for the eHL dimensions ranged from 1 to 4. The highest levels of agreement were observed in the dimensions “Feel safe and in control” (mean = 2.74) and “Understanding of health concepts and language” (mean = 2.46; Table 2). In contrast, the lowest levels of agreement were found in the dimensions “Using technology to process health information” (mean = 1.89) and “Access to digital services that work” (mean = 1.99; Table 2).

**Table 2:** Mean and standard deviation distribution of eHLQ responses (*n* = 101).

eHLQ Dimension	Minimum	Maximum	Mean	Standard deviation	Cronbach’s α (this sample)
Using technology to process health information	1.00	3.80	1.89	0.84	0.92
Understanding of health concepts and language	1.20	3.80	2.46	0.52	0.60
Ability to actively engage with digital services	1.00	4.00	2.11	0.92	0.92
Feel safe and in control	1.40	4.00	2.74	0.52	0.76
Motivated to engage with digital services	1.00	4.00	2.13	0.82	0.89
Access to digital services that work	1.00	3.50	1.99	0.64	0.81
Digital services that suit individual needs	1.00	3.75	1.98	0.83	0.91

Across the seven dimensions, mean scores ranged between 1.89 (Using technology to process health information) and 2.74 (Feeling safe and in control), suggesting modest levels of eHealth literacy overall. Standard deviations were generally wide, indicating considerable variability within the sample.

In this sample, the overall internal consistency of the eHLQ was excellent (Cronbach’s *α* = 0.95). At the dimension level, α values ranged from acceptable to excellent: Using technology to process health information (*α* = 0.92), Understanding of health concepts and language (*α* = 0.60), Ability to actively engage with digital services (*α* = 0.92), Feeling safe and in control (*α* = 0.76), Motivation to engage with digital services (*α* = 0.89), Access to digital services that work (*α* = 0.81), and Digital services that suit individual needs (*α* = 0.91). These findings support the reliability of the instrument in the present context (Table 2).

### 3.3 eHealth literacy and sociodemographic variables

No statistically significant differences were found in any of the eHLQ dimensions in relation to gender (Table 3).

**Table 3:** Significance of differences in eHLQ dimensions according to sociodemographic and professional variables.

Variables		(1)	(2)	(3)	(4)	(5)	(6)	(7)
		M (SD)	M (SD)	M (SD)	M (SD)	M (SD)	M (SD)	M (SD)
Gender	Male	1.95 (0.82)	2.41 (0.58)	2.18 (0.91)	2.72 (0.56)	2.16 (0.85)	2.01 (0.67)	2.10 (0.83)
	Female	1.83 (0.86)	2.50 (0.46)	2.04 (0.93)	2.76 (0.48)	2.10 (0.79)	1.98 (0.62)	1.88 (0.81)
	*Sig.*	0.497	0.386	0.453	,725	0.709	0.783	0.180
Age	18–24	2.78 (0.53)	2.55 (0.76)	2.92 (0.82)	2.60 (0.64)	2.88 (0.61)	2.58 (0.62)	2.65 (0.62)
	25–64	1.85 (0.79)	2.48 (0.49)	2.12 (0.84)	2.75 (0.48)	2.13 (0.75)	1.95 (0.60)	1.99 (0.80)
	> 64	1.13 (0.46)	2.23 (0.40)	1.18 (0.57)	2.80 (0.60)	1.28 (0.59)	1.65 (0.52)	1.21 (0.51)
	*Sig.*	0.001***	0.248	0.001***	0.562	0.001***	0.001***	0.001***
*Education*	None	1.06 (0.13)	2.32 (0.36)	1.10 (0.25)	2.60 (0.43)	1.36 (0.55)	1.58 (0.47)	1.13 (0.32)
	≤ First Cycle	1.14 (0.38)	2.27 (0.48)	1.29 (0.46)	2.84 (0.53)	1.50 (0.650)	1.56 (0.51)	1.26 (0.48)
	2nd e 3rd cycles	2.18 (0.69)	2.55 (0.42)	2.58 (0.70)	2.69 (0.48)	2.38 (0.64)	2.21 (0.59)	2.38 (0.64)
	Secondary	2.50 (0.61)	2.56 (0.68)	2.65 (0.72)	2.75 (0.59)	2.64 (0.59)	2.27 (0.58)	2.46 (0.66)
	*Sig.*	0.001***	0.091	0.001***	0.588	0.001***	0.001**	0.001***
Length of Stay in Portugal	< 1 year	1.95 (0.81)	2.27 (0.49)	2.00 (0.79)	2.65 (0.65)	2.28 (0.68)	1.88 (0.60)	2.07 (0.82)
	1–5 years	2.04 (0.66)	2.32 (0.51)	2.49 (0.68)	2.57 (0.46)	2.19 (0.66)	1.97 (0.58)	2.22 (0.58)
	6–9 years	2.70 (1.21)	2.85 (0.19)	2.50 (1.25)	2.55 (0.30)	2.60 (1.23)	2.38 (0.64)	2.19 (0.94)
	> 9 years	1.73 (0.86)	2.56 (0.52)	1.93 (0.99)	2.86 (0.48)	2.01 (0.88)	2.01 (0.67)	1.82 (0.90)
	*Sig.*	0.085	0.034*	0.066	0.081	0.510	0.575	0.210
Migration Status	Regular	1.88 (0.87)	2.53 (0.51)	2.14 (0.99)	2.83 (0.51)	2.11 (0.89)	2.09 (0.67)	2.01 (0.89)
	Irregular	2.24 (0.84)	2.36 (0.56)	2.31 (0.74)	2.64 (0.61)	2.31 (0.64)	1.85 (0.50)	2.11 (0.72)
	In regularization Process	1.76 (0.74)	2.32 (0.53)	1.93 (0.77)	2.52 (0.44)	2.08 (0.65)	1.77 (0.55)	1.85 (0.68)
	*Sig.*	0.033	0.194	0.397	0.031*	0.663	0.087	0.568

(1) “Using technology to process health information”; (2) “Understanding of health concepts and language”; (3) “Ability to actively engage with digital services”; (4) “Feel safe and in control”; (5) “Motivated to engage with digital services”; (6) “Access to digital services that work”; (7) “Digital services that suit individual needs”; M -Mean. SD – Standard Deviation; Sig. – Significance ^*^ *p* ≤ 0.05. ^**^ *p* ≤ 0.01. ^***^ *p* ≤ 0.001.

In terms of age, statistically significant differences were identified in the following dimensions:

- “Using technology to process health information “[*F* (2; 25.19) = 34.05, *p* = 0.001],
- “Ability to actively engage with digital services” [*F* (2; 22.11) = 20.54, *p* = 0.001],
- “Motivate to engage with digital services” [*F* (2; 21.85) = 21.15, *p* = 0.001],
- “Access to digital services that work” [*F* (2; 3.00) = 8.45, *p* = 0.001], and
- “Digital services that suit individual needs” [*F* (2; 23.58) = 20.62, *p* = 0.001; Table 3].

Regarding educational level, statistically significant differences were found in the following dimensions:

- “Using technology to process health information” [*F* (3; 51.31) = 62.38, *p* = 0.001],
- “Ability to actively engage with digital services” [*F* (3; 45.42) = 56.96, *p* = 0.001],
- “Motivate to engage with digital services” [*F* (3; 95) = 22.68, *p* = 0.001],
- “Access to digital services that work” [*F* (3; 95) = 11.44, *p* = 0.001], and
- “Digital services that suit individual needs” [*F* (3; 4.67) = 42.35, *p* = 0.001; Table 3].

Regarding length of stay in Portugal, a statistically significant difference was observed in the following dimension:

- “Understanding of health concepts and language” [*F* (3; 97) = 3.02, *p* = 0.034; Table 3].

With respect to migration status, statistically significant differences were found in the following dimension:

- “Feel safe and in control” [*F* (2; 98) = 3.61, *p* = 0.031; Table 3].

The *post hoc* analysis using Tukey’s multiple comparison test, considering the age groups, revealed significant differences in the dimensions “Using technology to process health information,” “Ability to actively engage with digital services,” “Motivate to engage with digital services” and “Digital services that suit individual needs.”

Participants aged between 18 and 24 years showed higher mean scores in these dimensions compared to those aged between 25 and 64 years, who, in turn, presented higher scores than participants aged 65 years or older.

In the dimension “Access to digital services that work,” participants aged between 18 and 24 years also reported higher mean scores than those in the 25–64 and >64 age groups (Table 3).

Bivariate analyses suggested differences in eHLQ scores by age, education, and chronic disease status. For example, younger participants (18–24 years) consistently reported higher scores in most dimensions compared with older age groups. Effect sizes ranged from small to moderate (*η*^2^ = 0.08–0.21), suggesting that age accounts for a meaningful proportion of variance in eHLQ outcomes.

Tukey’s test, applied to educational level, showed that participants with lower or upper secondary education or equivalent reported higher mean scores in the dimensions “Using technology to process health information,” “Ability to actively engage with digital services,” “Motivate to engage with digital services,” “Digital services that suit individual needs,” and “Access to digital services that work,” when compared to those with only primary education or no formal education (Table 3).

Participants with secondary education or higher also tended to score above those with only primary education or none, with medium-to-large effect sizes observed in several dimensions (Cohen’s d ranging from 0.50 to 0.90).

Regarding length of residence in Portugal, significant differences were found in the dimension “Understanding of health concepts and language,” with participants who had been living in the country for less than 1 year reporting significantly lower mean scores than those who had been residing in Portugal for more than 1 year (Table 3).

Finally, with respect to migration status, significant differences were observed in the dimension “Feel safe and in control.” Participants who were in the process of regularization reported lower mean scores compared to those with either regularized or irregular migration status (Table 3).

### 3.4 eHealth literacy and perceived health status

In order to assess the association between the dimensions comprising the eHLQ scale and perceived health status, Pearson’s correlation coefficient was used as a measure of linear association between quantitative variables (Table 4).

**Table 4:** Correlations between the eHLQ and perceived health status.

eHLQ dimensions	Perceived health status	
Using technology to process health information	p	0.424^**^
	r	0.001
Understanding of health concepts and language	p	0.096
	r	0.339
Ability to actively engage with digital services	p	0.472^**^
	r	0.001
Feel safe and in control	p	−0.002
	r	0.982
Motivate to engage with digital services	p	0.286^**^
	r	0.004
Access to digital services that work	p	0.269^**^
	r	0.007
Digital services that suit individual needs	p	0.432^**^
	r	0.001

Regarding the dimensions of the eHLQ scale, positive associations were found with the dimensions “Using technology to process health information” (*r* = 0.424), “Ability to actively engage with digital services” (*r* = 0.472), and “Digital services that suit individual needs” (*r* = 0.432), with the correlation coefficients indicating moderate strength. The dimension “Access to digital services that work” also showed a statistically significant and positive correlation with perceived health status (*r* = 0.286), with the correlation coefficient indicating weak strength. Thus, higher eHL in these dimensions is associated with better perceived health status (Table 4).

### 3.5 eHealth literacy and chronic diseases

The presence of chronic diseases was analyzed in relation to eHL. Statistically significant differences were found in the following dimensions:

- “Using technology to process health information” [*t* (98) = 4.26, *p* = 0.001],
- “Ability to actively engage with digital services” [*t* (99) = 4.29, *p* = 0.001],
- “Motivate to engage with digital services” [*t* (99) = 3.38, *p* = 0.001],
- “Access to digital services that work” [*t* (99) = 2.50, *p* = 0.007], and
- “Digital services that suit individual needs” [*t* (99) = 4.72, *p* = 0.001; Table 5].

**Table 5:** Significance of differences in eHLQ dimensions according to chronic disease status.

eHLQ dimensions	Chronic disease		No chronic disease		Sig.
	*M*	SD	*M*	SD	
Using technology to process health information	1.53	0.72	2.19	0.81	0.001***
Understanding of health concepts and language	2.50	0.50	2.43	0.54	0.250
Ability to actively engage with digital services	1.71	0.84	2.44	0.86	0.001***
Feel safe and in control	2.80	0.53	2.69	0.50	0.311
Motivate to engage with digital services	1.84	0.79	2.36	0.77	0.012**
Access to digital services that work	1.82	0.60	2.13	0.64	0.007**
Digital services that suit individual needs	1.61	0.79	2.29	0.73	0.001***

SD, Standard Deviation; Sig., Significance; *p ≤ 0.05, **p ≤ 0.01, ***p ≤ 0.001.

Migrant participants without chronic diseases reported significantly higher mean scores in the dimensions “Using technology to process health information” (*p* = 0.001), “Ability to actively engage with digital services” (*p* = 0.001), “Motivate to engage with digital services” (*p* = 0.012), “Access to digital services that work” (*p* = 0.007), and “Digital services that suit individual needs” (*p* = 0.001) when compared to those with chronic diseases (Table 5).

As shown in Table 5, participants with chronic diseases had significantly lower levels of digital health literacy in almost all dimensions of the eHLQ when compared to those without chronic diseases. This finding reinforces the need for interventions specifically targeting migrants with chronic diseases, who combine clinical and digital vulnerability.

## 4 Discussion

The interpretation of the present study’s findings should be approached with caution, as the exploratory nature of the research limits the possibility of drawing causal inferences. Nevertheless, the available literature provides a suitable basis for discussing the observed patterns and situating them in the broader context of eHL research.

The results of the present study, when compared with similar studies (28, 29), revealed lower levels of eHL, which may be associated with the different origins and cultural contexts of the populations under study. The dimensions “Feel safe and in control” and “Understanding of health concepts and language” showed the highest scores, which is consistent with the findings of Cheng et al. (28, 29), suggesting that participants demonstrate confidence in digital health systems as well as competencies in understanding health information.

In contrast, the dimensions “Using technology to process health information” and “Access to digital services that work” presented the lowest scores, which was also observed in the aforementioned studies (28, 29), reflecting limitations in the practical use of technology and difficulties in accessing effective and functional systems.

Considering the sample distribution in terms of gender, it aligns with the patterns reported in Portuguese public demographic data, which show no significant differences in the prevalence of migration patterns between genders (30). In this context, the results of the present study did not reveal statistically significant differences in any of the eHLQ dimensions, suggesting a homogeneous distribution of eHL levels between male and female participants.

These results contrast with those reported by García-García et al. (29), whose study, conducted among primary healthcare users in Spain, identified significant gender differences, with men presenting higher eHL levels in certain dimensions. On the other hand, Cheng et al. (28) support the findings of the present study, as they also did not identify gender-related variations.

Regarding the representation of different age groups, the sample is largely composed of a relatively young population of working and reproductive age, which reflects a common and global characteristic of most migratory flows. Age emerged as a determining factor in eHL, as participants aged between 18 and 24 years reported significantly higher mean scores in the dimensions “Using technology to process health information,” “Ability to actively engage with digital services,” “Motivate to engage with digital services,” “Digital services that suit individual needs,” and “Access to digital services that work”.

These results may be associated, on the one hand, with the greater digital familiarity and engagement typically seen among younger individuals and, on the other hand, with their more effective ability to access and use digital tools applied to the specific context of healthcare (29, 31).

Similarly, as evidenced in related studies (29), the results of this sample also confirm that older subgroups tend to present lower levels of eHL, particularly in dimensions related to navigation and the effective use of digital platforms. Additionally, it is known that digital difficulties among older adults often reflect lower technological proficiency and greater concerns about security and the privacy of health data (25, 32, 33).

Regarding the educational level of the participants, those with higher levels of education, specifically those holding lower and upper secondary education, reported higher mean scores in most eHLQ dimensions when compared to participants whose education was limited to primary school or who had no formal education. These results are consistent with existing literature, thus confirming a positive correlation between education and eHL (29, 34).

However, this finding was not confirmed in the dimensions “Understanding of health concepts and language” and “Feel safe and in control,” where no significant differences were observed in relation to educational level. Previous studies have also recognized that educational level showed a weak and negative correlation with the dimension “Feel safe and in control” (29, 35), suggesting that higher education does not necessarily translate into greater confidence or better understanding of health concepts and language. Nevertheless, other studies have shown that higher levels of education may be associated with lower confidence in digital technologies (33).

Regarding migration status, the present study showed that participants in the process of regularization reported lower mean scores in the dimension “Feel safe and in control” when compared to individuals with either regularized or irregular status. A study conducted in Portugal, which aimed to assess eHL among migrants with a focus on access, use, and trust in online health information (33), found that although the majority of migrants had access to digital services, approximately 45.6% expressed distrust regarding the reliability of online health information. This was particularly evident among participants with lower incomes and those who had recently arrived in the country.

This association seems to reinforce the idea that factors linked to migration status, such as language barriers and cultural adaptation, may contribute to a lower perception of safety and control when accessing and using digital health services.

In this regard, the dimension “Understanding of health concepts and language” showed significant differences in relation to length of residence in Portugal. However, despite the study population being fluent in Portuguese and mostly originating from Portuguese-speaking African countries, participants who had been living in Portugal for less than 1 year reported significantly lower mean scores when compared to those who had been residing in the country for more than 1 year.

Thus, the duration of stay in the host country often reflects significant differences in this respect (36), which may not be exclusively related to linguistic comprehension of the host country’s language. Supporting this idea, it is considered that, in addition to language proficiency, factors such as prior digital literacy, educational background, and familiarity with digital health systems in the countries of origin play a key role in eHL (28, 35). Therefore, length of residence alone does not appear to be a determining factor in eHL, making it necessary to consider other variables.

The results of the present study demonstrated a positive correlation between eHL and perceived health status, supporting previous evidence that points to an association between higher levels of eHL and more positive self-perceptions of health (2, 23). The dimensions “Ability to actively engage with digital services,” “Digital services that suit individual needs,” and “Using technology to process health information” showed moderate correlations, which seems to suggest not only greater participant engagement in managing their own health but also greater self-efficacy in using digital health resources. In turn, the dimension “Access to digital services that work” revealed a significant but weak correlation, further reinforcing the facilitating role of technology in accessing healthcare, in line with the findings of Kayser et al. (23). Conversely, the absence of an association with the dimension “Feel safe and in control” may reflect the influence of cultural, sociodemographic, or contextual factors specific to the migrant population, as highlighted in the literature (29, 31).

Concerning internet use for health purposes, some studies show that not all internet use leads to better health behaviors (37, 38), particularly when it involves sharing health-related content on social media or via email (37). Actively seeking health information online may be protective; however, the quality of internet use seems to matter more than the frequency of use. In this sense, frequent use of the internet for health purposes has been associated with maintaining or improving health, although higher general internet use has also been linked to poorer health status over time. Thus, frequent internet use among individuals with limited health knowledge does not necessarily indicate higher eHL, nor is it necessarily associated with better health outcomes.

In relation to the presence of chronic disease and its correlation with eHL levels, the results of the present study revealed statistically significant differences in most eHLQ dimensions, aligning with the available literature (11, 39, 40), while also considering the role of internet use in this relationship (8, 37). In particular, participants with chronic disease recorded lower mean scores in most of the dimensions assessed by the eHLQ, which is consistent with previous studies (41, 42).

Although higher levels of eHL appear to be broadly associated with greater readiness to adopt and maintain healthy behaviors (37), this association does not seem to be as consistent among individuals with chronic disease. A study by Lee and Tak (34) presented opposite results, identifying higher levels of eHL among people with at least one chronic disease compared to healthy individuals. The authors attributed this difference to the greater exposure of people with chronic disease to digital health systems, as a result of more frequent use.

From this perspective, the data suggest that the relationship between eHL and overall health may differ from the relationship between eHL and the presence of chronic diseases. This finding highlights the need to consider other health determinants, particularly given the diversity of migrant populations.

With regard to the motivation factor, the dimension “Motivate to engage with digital services” was lower among participants with chronic disease than among those who did not report having a chronic health condition. This contrasts with another study (43), which reports greater motivation to use eHealth among individuals with chronic diseases, also highlighting an increase in motivation proportional to the complexity of the disease.

A meta-analysis by Kim et al. (40) demonstrated a moderate positive correlation between eHL and health-promoting behaviors, suggesting that higher levels of eHL are associated with better health choices. However, this association was less pronounced among participants with chronic disease, further reinforcing this specific distinction.

In the context of eHL among individuals with chronic disease, several explanations can be proposed, once again pointing to more frequent contact with healthcare professionals and the fact that chronic disease management requires more than just access to information. Motivational and social factors, access to healthcare, and individual and cultural conceptions of health and illness should also be considered.

When analyzing the results through the lens of the “Transactional Model of eHealth Literacy” (10), the sample shows reduced levels of eHL in the dimensions where a more practical and active component predominates, such as access to and use of digital health services. This may reflect limitations in the transactional dynamics described by the model, pointing to constraints in contextual support. Indeed, the data reveal high levels of confidence and understanding of health concepts, even in the presence of overall low levels of eHL, suggesting the existence of an underlying cognitive and motivational capital. Although the eHLQ was developed based on the “eHealth Literacy Framework” (19), its multiple dimensions appear to capture aspects of the dynamic interactions between the individual and the digital environment, aligning with the approach proposed by the “Transactional Model of eHealth Literacy.”

This analysis reinforces the need for metrics and strategies that act simultaneously on both the individual and the context, taking into account the dynamic nature of both the digital environment and the migratory context.

### 4.1 Implications for clinical practice, training, and health policymaking

The findings of this study underline the need for a multidimensional response to improve eHL among migrant populations. In clinical practice, professionals should recognize that patients with low eHL (particularly those living with chronic conditions) require adapted communication strategies, clear explanations, and practical demonstrations when digital tools are introduced. The use of cultural mediators and community health workers may be critical to facilitate trust, overcome linguistic or cultural barriers, and promote adherence to digital health interventions.

In the field of training, curricula for health professionals should incorporate modules on digital health communication, health literacy, and cultural competence. Developing these skills is essential to empower healthcare teams to support diverse populations in navigating digital environments.

From a policy perspective, efforts should go beyond individual-level interventions to address structural barriers. Investment in community-based digital literacy programs, tailored to migrants’ linguistic and cultural needs, can help bridge gaps in access. At the same time, promoting inclusive eHealth design with multilingual interfaces, simplified navigation, and participatory co-design with users, can enhance usability and reduce risks of digital exclusion. Such approaches would not only improve equity in access to health information and services but also contribute to more sustainable and culturally sensitive healthcare systems.

### 4.2 Limitations

Several methodological limitations should be acknowledged. First, the use of a non-probabilistic convenience sample may have introduced selection bias, limiting the generalizability of findings to broader migrant populations. Second, the exclusion of non-Portuguese speakers, although necessary to ensure comprehension of the questionnaires, likely underestimated the magnitude of digital health disparities, as language barriers represent a major determinant of eHL. Third, chronic disease status was self-reported, raising the possibility of recall bias or underreporting compared with medically verified diagnoses. Fourth, the analyses were restricted to descriptive and bivariate approaches. Although effect sizes were reported, the absence of multivariable models prevented adjustment for potential confounders such as age, education, or length of residence in Portugal. Consequently, some associations, for example the link between chronic disease and lower eHL, may be partly explained by these unmeasured factors.

Finally, the relatively small sample size increases the risk of type II error and limits the precision of estimates. These limitations highlight the exploratory nature of the study and reinforce the need for future research with larger, more diverse samples, multilingual instruments, and robust analytical strategies.

### 4.3 Suggestions for future research

Future research should further explore the intersection between eHL, migration, and chronic disease management across different sociocultural contexts. Longitudinal studies are needed to better understand causal relationships between eHL and health outcomes in migrant populations. Comparative studies between migrant groups from different countries of origin would also provide insights into the role of cultural background in shaping digital health engagement.

Additionally, qualitative studies could deepen the understanding of migrants lived experiences with digital health, uncovering barriers such as trust, privacy concerns, and usability of digital platforms. Intervention studies should also be designed and tested to evaluate the effectiveness of culturally tailored digital training programs and their impact on improving both eHL and health outcomes.

Finally, future investigations should consider the role of structural determinants, such as migration status, housing, and employment conditions, as mediators of eHL, thus supporting the development of more comprehensive health equity policies.

## 5 Conclusion

This study provides exploratory evidence of the association between health status and weaknesses in digital competencies faced by a migrant community, highlighting the intersection between chronic disease and eHL. Given its cross-sectional and convenience-sampling design, findings should be interpreted with caution, as representativeness and causal inference are limited. Nevertheless, in a context marked by rapid technological development and the digitalization of health resources, the coexistence of low eHL and chronic health conditions emerges as a critical concern for vulnerable groups such as migrant populations.

These results reinforce the need to promote eHL in a culturally and linguistically contextualized manner, accounting for literacy levels, language proficiency, and sociocultural specificities of the target population. Tailored strategies (including in-person support, accessible communication, inclusive digital design, and the use of cultural mediators) may help mitigate inequalities and foster equitable access to digital health services.

Importantly, the exploratory nature of this study highlights the urgent need for future longitudinal and intervention research to clarify causal pathways and evaluate the effectiveness of tailored digital health programs. Longitudinal approaches could determine how eHL influences health trajectories over time, while intervention studies could assess the impact of culturally adapted digital training and inclusive eHealth strategies on health outcomes.

Ultimately, investing in culturally and linguistically tailored digital health strategies, supported by robust empirical evidence, represents a key step toward reducing disparities and building more equitable healthcare systems for migrant populations.

## Data Availability

The original contributions presented in the study are included in the article/supplementary material, further inquiries can be directed to the corresponding author.
